# Ambiguities About Discussing End of Life in the Context of Advance Care Planning in Dementia

**DOI:** 10.1093/geroni/igaf122.2601

**Published:** 2025-12-31

**Authors:** Jenny van der Steen, Lieve Van den block, Miharu Nakanishi, Jürgen in der Schmitten, Rebecca L Sudore, Ida J Korfage

**Affiliations:** Leiden University Medical Center, Leiden, Zuid-Holland, Netherlands; Vrije Universiteit Brussel, Brussels, Brussels Hoofdstedelijk Gewest, Belgium; Tohoku University Graduate School of Medicine, Sendai, Miyagi, Japan; Universitätsklinikum Essen, Essen, Nordrhein-Westfalen, Germany; University of California, San Francisco, San Francisco, California, United States; Erasmus University Medical Center, Rotterdam, Zuid-Holland, Netherlands

## Abstract

Advance care planning (ACP) in dementia has been defined as a communication process adapted to the person’s capacity, which includes, and is continued with family, in our European Association for Palliative Care Delphi study Delphi study with an expert panel from 33 countries. A consensus was achieved that ACP discussions can refer to the end of life, but it is not a requirement. The study comprised four survey rounds and offered ample room for qualitative feedback. The feedback indicated ambiguity around discussing the end of life in the context of ACP. Regardless of a consensus-according to conservative criteria-to offer ACP around dementia diagnosis and to raise end-of-life issues later, there were concerns that bringing up the end of life could cause emotional harm to the person with dementia and the family. The panelists suggested that adopting a tailored, person-centered approach to ACP could help prevent such harms. On the other hand, there were concerns that being too careful could result in missed opportunities to talk about the end of life and elicit the persons’ preferences when still able. Sketching a realistic scenario of the future and its uncertainties, probing attitudes of the person and their family, and offering choices may prevent possible detrimental effects of initiating ACP and discussing the end of life even early on. Taking such an individualized approach is consistent with the understanding of ACP as part of a process rather than a one-time event and may lower barriers to initiate ACP.

